# Differential Expression of Matrix Metalloproteinase-2 Expression in Disseminated Tumor Cells and Micrometastasis in Bone Marrow of Patients with Nonmetastatic and Metastatic Prostate Cancer: Theoretical Considerations and Clinical Implications—An Immunocytochemical Study

**DOI:** 10.1155/2012/259351

**Published:** 2012-11-26

**Authors:** Nigel P. Murray, Eduardo Reyes, Pablo Tapia, Leonardo Badínez, Nelson Orellana

**Affiliations:** ^1^Hematology, Division of Medicine, Hospital de Carabineros de Chile, Simón Bolívar 2200, Ñuñoa, 7770199 Santiago, Chile; ^2^Instituto de Bio-Oncología, Avenida Salvador 95, Oficina 95, Providencia, 7500710 Santiago, Chile; ^3^Circulating Tumor Cell Unit, Faculty of Medicine, Universidad Mayor, Renato Sánchez 4369, Las Condes, 7550224 Santiago, Chile; ^4^Faculty of Medicine, Universidad Diego Portales, Manuel Rodriguez Sur 415, 8370179 Santiago, Chile; ^5^Faculty of Medicine, Universidad Pontificia Católica de Chile, Avenida Libertador Bernardo O'Higgins 340, 8331150 Santiago, Chile; ^6^Radiotherapy, Fundación Arturo López Pérez, Rancagua 899, Providencia, 7500921 Santiago, Chile

## Abstract

Matrix metalloproteinase-2 (MMP-2) is important in the dissemination and invasion of tumor cells and activates angiogenesis. We present an immunocytochemical study of MMP-2 expression in circulating prostate cells (CPCs), disseminated tumor cells (DTCs), and micrometastasis (mM) in bone marrow of men with prostate cancer. *Methods and Patients*. Tumor cells were identified with anti-PSA immunocytochemistry. Positive samples underwent processing with anti-MMP-2, its expression was compared with Gleason score, concordance of expression, and metastatic and nonmetastatic disease. *Results*. 215 men participated, CPCs were detected in 62.7%, DTCs in 62.2%, and mM in 71.4% in nonmetastatic cancer; in metastatic cancer all had CPCs, DTCs, and mM detected. All CPCs and DTCs expressed MMP-2; in mM MMP-2 expression was positively associated with increasing Gleason score. MMP-2 expression in CPCs and DTCs showed concordance. In low grade tumors, mM and surrounding stromal cells were MMP-2 negative, with variable expression in high grade tumors; in metastatic disease, both mM and stromal cells were MMP-2 positive. *Conclusions*. CPCs and DTCs are different from mM, with inhibition of MMP-2 expression in mM of low grade tumors. With disease progression, MMP-2 expression increases in both mM and surrounding stromal cells, with implications for the use of bisphosphonates or MMP-2 inhibitors.

## 1. Introduction

With the increasing use of prostate specific antigen as a screening test to detect prostate cancer, the frequency of men presenting with metastatic disease has decreased [1, 2]. However, the death rate from prostate cancer has only slightly fallen [3], with metastatic disease being the commonest scenario leading to death. At least 85% of men with advanced disease will have bone metastasis [4, 5], with an increasing number of these patients believed to be metastasis-free at the time of initial treatment but who had occult micrometastasis. Furthermore, 30% to 50% of men with localized prostate cancer will develop biochemical failure with an increased PSA at 10 years. This is due to dissemination of cancer cells early in the disease and being not detected by conventional methods.

Cancer cells disseminate from the original cancer, first to the neurovascular structures and then to the blood [6]. From there they pass to other tissues where they may pass through (cells in transit) or adhere to the capillary endothelium and invade, forming micrometastasis. Tumor invasion is considered to be an unregulated physiological activity, with similarities between the molecular events of tumor invasion and normal processes such as angiogenesis and wound healing. One common denominator is the involvement of the matrix metalloproteinases. These are endopeptidases capable of degrading the extracellular matrix, contain zinc in their structure, and are secreted in latent form and later activated. It is postulated that they have an important role in metastasis and the liberation of growth factors [7, 8]. Matrix metalloproteinase-2 (MMP-2) is a gelatinase and its expression has been reported to be increased in prostate cancer [9–11]. There is an association between MMP-2 expression in the primary tumor and the Gleason score, pathological stage, and as an independent prognostic factor [11, 12]. In addition MMPs have been shown to be involved in the release of growth factors that enhance tumor growth and aggressiveness [13–15].

If, as the reports indicate, increased MMP-2 expression in the primary tumor is associated with a worse prognosis, one explication could be that cells expressing MMP-2 disseminate early to distant tissues, are not therefore affected by loco-regional treatments, and as a consequence are able to develop into metastasis. If this hypothesis is correct, circulating tumor cells should express MMP-2 whether they are circulating in blood or the bone marrow, and MMP-2 expression would permit the invasion of the endostium and therefore facilitate the formation of micrometastasis. The coexpression of PSA and MMP-2 in bone marrow fragments would confirm this hypothesis.

These data prompted us to investigate the expression of MMP-2 in circulating prostate cells in blood and bone marrow, and in the micrometastasis in bone marrow fragments in a population of patients with prostate cancer, after radical prostatectomy, both in patients bone scan negative and positive.

## 2. Methods and Patients

The transverse population included patients diagnosed with prostate cancer attending the Hospital de Carabineros de Chile and the Instituto of BioOncología, Santiago, Chile between 2008 and 2011. Patient records were used to retrieve clinical information (age, stage, Gleason score, treatment, bone scan results, and serum PSA at the time of sampling).

The following is definition of circulating prostate cells (CPCs), disseminated tumor cells (DTCs) in bone marrow aspirates, and micrometastasis (mM). The criteria of ISHAGE were used to evaluate immunostained cells [16].

**Figure 1:**
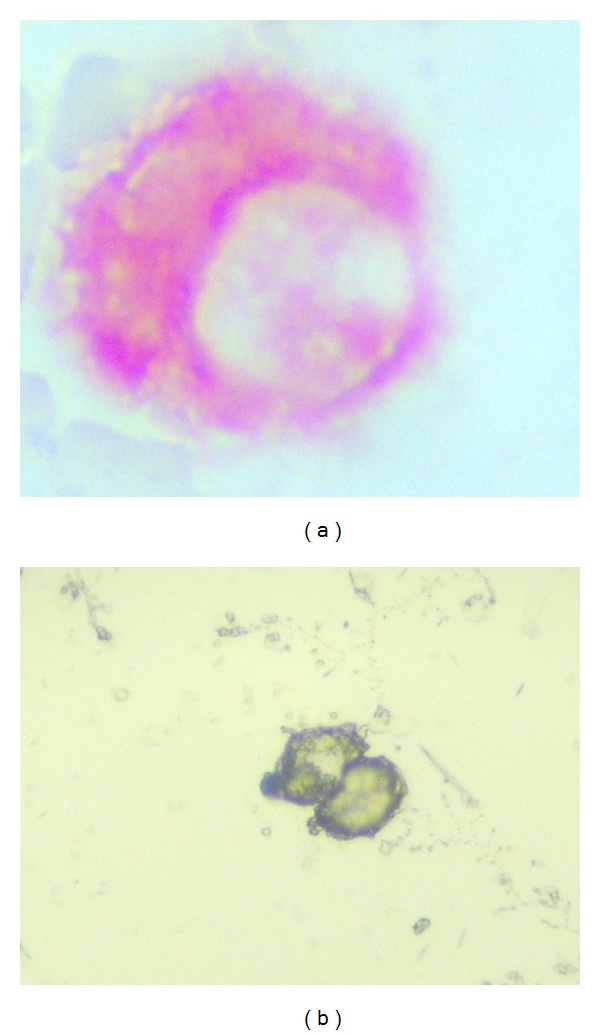
(a) PSA (+) CPC. (b) circulating leucocytes.

**Figure 2:**
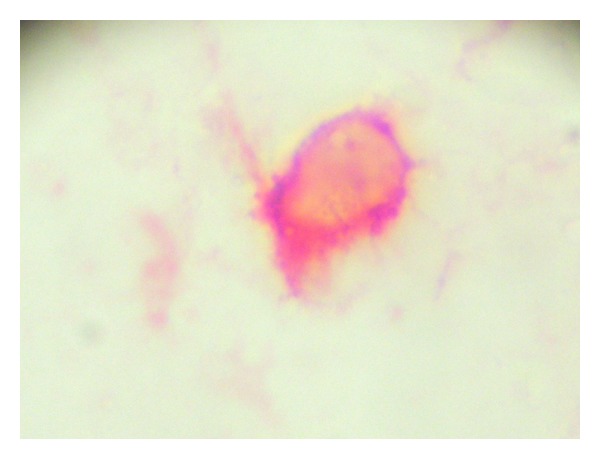
Disseminated tumor cell.

**Figure 3:**
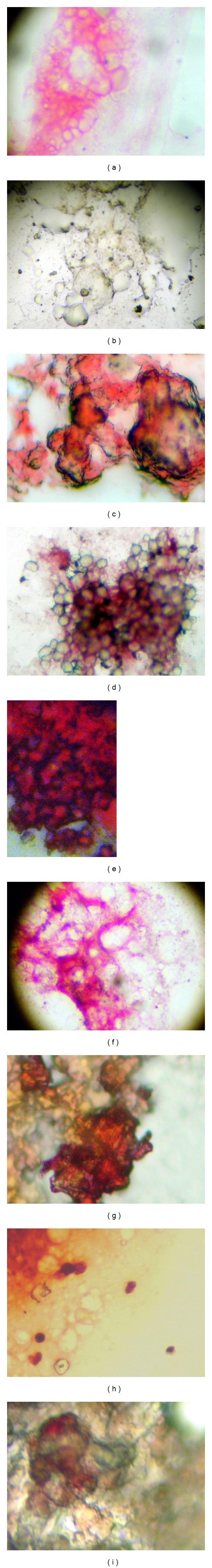
(a) Micrometastasis PSA (+). (b) Biopsy PSA (−). (c) Borders of microfragment MMP-2 (+). (d) Central pattern MMP-2 (+) stromal cells MMP-2 (+). (e) Central pattern MMP-2. (f) Microfragment MMP-2 (−), surrounding stromal cells MMP-2 (−). (g) Borders of microfragment MMP-2 (+), surrounding stromal cells MMP-2 (−). (h) Microfragment MMP-2 (−), stromal cells MMP-2 (−), DTC MMP-2 (+). (i) Borders of microfragment MMP-2 (+), some stromal cells MMP-2 (+).

- CPCs: secondary CPC: detected in blood after radical treatment (Figure 1(a)).
- DTCs: cells detected in bone marrow aspirates or bone marrow biopsy touch preparations, but not in bone marrow fragments (Figure 2).
- Micrometastasis: cells detected in bone marrow fragments from biopsy specimens (Figures 3(a)–3(i)).

### 2.1. Inclusion Criteria

- Biopsy proven prostate cancer.
- Written informed consent.
- Bone scan within three months of the sampling.

### 2.2. Sample Preparation

After written informed consent bone marrow samples were obtained by an aspiration (4 mL) and a biopsy from the posterior superior iliac crest, and an 8 mL venous blood sample was taken at the same time.

#### 2.2.1. Blood and Bone Marrow Aspiration

4 mL aspirate sample of bone marrow and 8 mL of blood were collected into EDTA (Beckinson-Vacutainer) and processed within 30 minutes. The sample was layered onto 2 mL Histopaque 1.077 (Sigma-Aldrich) at room temperature. The mononuclear cells were obtained according to manufacturer's instructions and finally washed 3 times in phosphate buffered saline pH 7.4 (PBS). The pellet was resuspended in 100 *μ*L of autologous plasma and 25 *μ*L was used to prepare each slide (silanized DAKO, USA). The slides were air-dried for 24 hours and finally fixed in a solution of 70% ethanol, 5% formaldehyde and 25% PBS for 5 minutes and then washed 3 times with PBS.

#### 2.2.2. Biopsy

The bone marrow biopsy sample was used to make 3 “touch-preps” using silanized slides (DAKO, USA) and fixed as previously described.

### 2.3. Immunocytochemistry

Monoclonal antibodies directed against PSA clone 28A4 (Novacastro, UK) in a concentration of 2,5 *μ*g/mL were used to detect prostate cells and identified using a detection system based on alkaline phosphatase-antialkaline phosphatase (LSAB2 DAKO, USA) with new-fuchsin as the chromogen. To permit the rapid identification of positive cells there was no counter staining with Mayer's hematoxilin. Levisamole (DAKO, USA) was used as an inhibitor of endogenous alkaline phosphatase. Positive and negative controls were processed in the same way.

Positive samples underwent a second stage of processing, using the monoclonal antibody against MMP-2 clone 1B4 (Novocastra, UK) and a system of detection based on peroxidase (LSAB2, DAKO, USA) with DAB (DAKO, USA) as the chromogen. Endogenous peroxidase was inhibited using an inhibitor (DAKO, USA) according to the manufacturer's instructions.

### 2.4. Definition of Expression of MMP-2

The criteria to define a cell expressing MMP-2 were that of Trudel et al. (2003) [12], con the percentage of PSA positive cells coexpressing MMP-2 grouped as 0%, 1–10%, 11–50%, and >50%, and the cells were additionally classified semiquantitatively as having 0, 1+, 2+, 3+ intensity of immunostaining (see Figure 1). A mean MMP-2 score was calculated for each sample, defined as total MMP-2 expression/N° of cells. In the samples of bone marrow biopsy touch preps the expression of MMP-2 in the surrounding non-PSA expressing cells was analyzed. The expression in these cells was noted as present or absent and the intensity of MMP-2 expression was noted. In blood and bone marrow aspirate samples this was not assessed as due to the nature of cell separation, and cells in the stained stain have no relation between each other.

Samples were analyzed at low power and photographed at a magnification of 400x using a digital camera, Samsung Digimax D73, and processed with the Digimax program for Windows 98. The immunocytochemical evaluation was performed by a single person, blinded to the clinical details using a coded system.

The patients were divided into 2 groups:

- postradical prostatectomy and bone scan negative, with or without biochemical failure,
- postradical prostatectomy bone scan positive with evidence of biochemical failure, defined as a PSA > 0.2 ng/mL in patients after radical prostatectomy.

### 2.5. Statistical Analysis

Descriptive statistics were used for demographic variables, expressed as mean and standard deviation in the case of continuous variables with a normal distribution. In case of an asymmetrical distribution the median and interquartile range (IQR) values were used. Noncontiguous variables were presented as frequencies. The Student's *t*-Test was used to compare continuous variables with a normal distribution, chi-squared, Kruskal-Wallis and log regression for the differences in frequency. The kappa test was used for tests of concordance.

### 2.6. Ethical Considerations

The study was directed with complete conformity with the principles of the declaration of Helsinki and approval of the local ethical committees.

## 3. Results

185 men bone scan negative and 30 men bone scan positive participated in the study. The presence of circulating prostate cells in bone marrow aspirates as well as in bone marrow of prostate cancer patients was analyzed by determining PSA protein expression. CPCs were detected in 62.7%, DTCs in 62.2%, and mM in 71.4% of patients. All men bone scan positive had CPCs, DTCs, and mM detected in 100% of the cases (Table 1).

PSA protein expression in cells present in blood, bone marrow aspirate (BMA), and biopsy of cancer patients was compared with the Gleason score in patients without evidence of micrometastatic disease. There was no difference in the detection of cells in relation to age or serum PSA levels or the time from diagnosis to test time. Patients with higher Gleason scores had significantly higher stage disease. There were no differences in the frequency of detection of CPCs and DTCs with regards to Gleason score; however, the frequency of detection of mM was significantly lower in patients with Gleason 4 in comparison with higher Gleason scores (Kruskal-Wallis, *P* < 0.001) (Table 2).

PSA protein expression in cells present in blood, bone marrow aspirates (BMA), and biopsy of cancer patients with macrometastasis was compared with the Gleason score. There were no significant differences in the frequency of detection of CPCs, DTCs or mM with regard to Gleason score or relation to the serum PSA at the time of sampling (Table 3).

The expression of matrix metalloproteinase-2 (MMP-2), in patients positive for prostate cells in blood (*n* = 116), bone marrow aspirates (*n* = 115), and bone marrow biopsy (*n* = 132) of men without metastatic disease showed that MMP-2 expression was commonly limited to the edge of the bone marrow fragment (Figure 3(c)). Men with higher Gleason scores had a significantly higher frequency of MMP-2 expression in the mM (chi squared for trends, *P* = 0.031), and all CPCs and DTCs expressed MMP-2 (Table 4).

There was concordance in MMP-2 expression between CPCs and DTCs but not with mM for all Gleason scores in men with nonmetastatic cancer.

In men with metastatic disease MMP-2 expression was present in all CPCs and DTCs as well as mM but was expressed in all parts of the bone marrow fragment, defined as central expression (Figures 3(d) and 3(e)). There was concordance between CPCs, DTCs, and mM for all Gleason scores for the expression of MMP-2 (Table 5).

Stromal cell expression of MMP-2 was variable, and in the majority of microfragments MMP-2 negative the stromal cells were also negative (Figure 3(f)). In those microfragments with borders positive for MMP-2 the stromal cells were usually MMP-2 negative (Figure 3(g)) but in one case of Gleason 9 some of the surrounding stromal cells were MMP-2 positive (Figure 3(i)). In samples of microfragments and stromal cells MMP-2 negative, DTCs nearby were MMP-2 positive (Figure 3(h)). Stromal cells surrounding microfragments centrally expressing MMP-2 also expressed MMP-2 (Figure 3(d)) (see Table 6).

## 4. Discussion

MMP-2 is one of a family of enzymes that cleave a broad range of components of the extracellular matrix (ECM), basement membrane, growth factors, and cell surface receptors [17, 18]. MMPs are upregulated in cancer progression, can act as oncogenes, and promote invasion and metastasis in virtually all solid tumors [17, 18]. These enzymes play a role not only in tumor initiation and invasion but also in angiogenesis, metastasis and in releasing other tumor-promoting factors. Stromal and inflammatory cells in the primary tumor, rather than tumor cells, typically synthesize MMPs, which can then act on the stroma and regulate the tumor microenvironment as well as act on tumor cells themselves [17, 18]. A key role in this process is carried out by integrins, a widespread family of ECM-specific cell surface receptors. Integrins are major mediators of both cell-ECM interactions and transduction of matrix generated signals regulating cell proliferation, motility, and apoptosis. In human breast carcinoma cells it has been shown that alpha5-beta1 integrin promotes invasion of breast carcinoma cells by upregulating MMP-2 activity [19]. Likewise tumor cell extravasation is a critical step in metastasis, studies show that this is an active [20, 21] and not a passive process driven by mechanical factors as first thought [22]. It is characterized by orchestrated signaling events involving adhesion molecules and cytokines, and the binding of and activation of MMP-2 promote tumor cell transmigration across the endothelial barrier and thus invade the distant tissue [23].

We believe that this is the first paper to describe the expression of MMP-2 in CPCs, DTCs, and mM. That both CPCs and DTCs express MMP-2 is consistent with the theory of the role of MMP-2 in the metastatic process of dissemination that cells expressing MMP-2 are able to penetrate the basement membrane and spread via the blood. That there is no association with the clinical parameters is in agreement with studies on prostate tissues [12], but also implies that only cells expressing the metalloproteinase have the inherent capacity to migrate.

There is a differential expression of MMP-2 in bone marrow micrometastasis, where the presence of MMP-2 detected by immunocytochemistry is in almost all cases zero in low grade cancer and suggests the inhibition of MMP-2. That the stromal microenvironment plays a critical role in determining tumor cell behavior has been shown in primary tumors [24, 25], where stromal cells increase MMP-2 expression in tumor cells. We describe, for the first time in prostate cancer, that bone marrow stromal cells produce the opposite reaction, that of inhibition of MMP-2 expression. The stromal cells surrounding the micrometastasis do not express MMP-2 in those cases where the micrometastasis is MMP-2 negative, stromal cell MMP-2 expression is variable when the micrometastasis has borders expressing MMP-2 and is stromal cell MMP-2 expression is positive when the micrometastasis has central expression of MMP-2. This might suggest that the inhibitor or inhibitors affect both stromal and tumor cell MMP-2 expression. In the case of border positive micrometastasis the variable stromal cell expression of MMP-2 may be explained by possible tumor cell factors stimulating the production of MMP-2 in both tumor and stromal cells. Thus stromal cell MMP-2 expression maybe a consequence of tumor cell activity. It has been shown that ANT2 shRNA suppresses induced migration and invasion by depletion of HER2/neu protein and, in turn, suppression of HER2/PI3 K/Akt pathway signaling and subsequent suppression of proteolytic activity by downregulating the activity of metalloproteinases [26]. Men with metastasis frequently have been treated previously with androgen blockade; androgen blockade has been shown to select prostate tumor cells which express HER-2 [27]. We suggest that it may be possible that micrometastasis from higher grade tumors or those micrometastases exposed previously to androgen blockade have a higher expression of HER-2 protein; this in turn leads to higher MMP-2 expression with increased invasion, secondary dissemination and finally angiogenesis and macrometastasis formation.

That the expression in mM is different to that in CPCs and DTCs is a supportive evidence that prostate cells detected in bone marrow aspirates are different and are not true micrometastasis [28], but represent circulating tumor cells in the bone marrow compartment. It has been shown that the bone marrow microenvironment is composed of specific niches that provide support for the proliferation and maintenance of hematopoietic stem cells [29], and interactions between the stem cells and their microenvironment regulate their maintenance, proliferation, differentiation, and migration into the blood circulation. Distinct niches have been anatomically and physiologically defined within the bone marrow [30, 31]. In the endosteal region, osteoblasts and other mesenchymal-derived stromal cells such as reticular cells, fibroblasts, and adipocytes constitute the osteoblastic niche that supports the maintenance of hematopoietic stem cells in a quiescent and undifferentiated state, by adhesion and humoral factors [32].

We propose that the expression of MMP-2 is inhibited by bone marrow stromal cells, in a process similar to that seen with hematopoietic stem cells, possibly by TIMP-2, although that other inhibitors modulate this function cannot be ruled out.

The inhibition of MMP-2 decreases the ability of the cancer to migrate from its new site, but does not inhibit proliferation directly. However, the decreased release of growth factors produced by MMP-2 and decreased initiation of angiogenesis by MMP-9 induced in part by MMP-2 [33] may limit the microfoci's growth potential. However, in high grade cancer, such as Gleason 9, tumor cells in micrometastasis continue to proliferate; as they divide and expand towards the intertrabecular surface, the inhibition by stromal cells decreases. This permits the reappearance of MMP-2 expression, as seen in the microfragment borders but not in the centre of the fragment, where MMP-2 suppression continues, which in turn allows the cell to escape and to disseminate, forming 2° CPCs.

In men with bone scan positive prostate cancer the expression of MMP-2 is throughout the bone fragment and involves the surrounding stromal tissue. These men had been previously treated with standard androgen blockade, and the overexpression of HER-2 protein caused by prior androgen blockade could increase MMP-2 expression and as a consequence MMP-2 is found throughout the microfragment (central pattern) and the surrounding stromal cells. Thus there may be two mechanisms involved in MMP-2 expression seen in the microfragments, firstly a passive phenomenon caused by cell proliferation towards the intertrabecular space resulting in decreased suppression of MMP-2 and secondly in more advanced disease, an active mechanism whereby HER-2 coexpression increases MMP-2 expression.

There is evidence for tumor-stroma crosstalk at metastatic sites; Kaminski et al. [34] described the effect of metastatic prostate cancer cell lines and nonprostatic stromal fibroblasts which are encountered by metastatic cells at most sites. For continuous growth and propagation at metastatic sites tumor cells have to induce a supportive stroma. Media conditioned by metastatic cell lines are able of inducing cultured fibroblasts to proliferate which corresponds to fibrous stroma induction *in vivo* [34]. Media conditioned by DU-145 metastatic prostate cell line can induce in fibroblasts the expression of MMP-14 mRNA, although other factors such as bFGF, PDGF, and TNF-alpha are also secreted in low amounts by DU-145 cells and they also stimulate the production of MMP-14 mRNA, possibly by activation of the Ets-1 transcription factor [35]. This crosstalk between stromal and cancer cells would explain the differences in MMP-2 expression found in the three groups of patients. Firstly the stromal cells inhibit MMP-2 expression, then with cancer progression the cancer cells induce stromal expression of MMP-2, which in turn leads to the release of other growth factors and angiogenic factors [36, 37].

This process may have important clinical implications; firstly the differential expression of MMP-2 between circulating cells and micrometastasis could explain the early dissemination of cancer cells through mechanisms mediated for MMP-2; having invaded the bone, the inhibition of MMP-2 has a direct effect of trapping the cancer cell in its new environment and through indirect processes limits its growth in terms of the size of the focus (bone scan negative), thus could explain why although bone marrow micrometastasis are frequent, local gross recurrence is more common than metastatic relapse [38], the microfoci entering a state of dormancy. In cancer cells that are proliferating in the bone marrow, for mechanical reasons, the cells grow into the intertrabecular space and as a result the inhibition of MMP-2 decreases and secondary dissemination is possible.

In patients with macrometastasis, that is, bone scan positive patients, these inhibitory mechanisms have been overcome, possibly by HER-2 overexpression, and MMP-2 expression is found throughout the bone marrow fragment; this in turn permits activation of the physiological mechanism previously mentioned, angiogenesis and growth of the secondary tumor.

Secondly, one of the mechanisms of action of the bisphosphonates is inhibiting MMP-2 through MMP-14 (MMP-MT1), if as we have shown that micrometastases do not express MMP-2. This may explain why clinical studies of bisphosphonates in prostate cancer patients have shown conflicting results in bone scan negative patients [39]. Thus inhibition of MMP-2 by BFs would decrease dissemination and infiltration of circulating cells, but would not affect the established micrometastasis. They may prevent or delay the appearance of MMP-2 expression thus delaying the formation of macrometastasis. However, in men with macrometastasis, with a positive bone scan the use of bisphosphonates may have a better therapeutic effect for the increased MMP-2 expression. Thus the role of bisphosphonates would have two different roles depending on the presence of micro- or macrometastatic disease.

## Figures and Tables

**Table 1:** Demographic characteristics of the study group.

	Group 1	Group 2
Patient number (*n*)	185	30
Mean age ± SD (years) at sampling	72.2 ± 9.0	76.4 ± 8.7
Median serum PSA (IQR) (ng/mL) at sampling	1,32 (0,01–5,77)	43,81 (27,72–150)
Median Gleason score at diagnosis (IQR)	6 (5–7)	6 (5–7)
Median stage at diagnosis (IQR)	3 (2-3)	3 (2-3)
Median time from diagnosis (IQR) (years)	3 (1–7)	6 (4–9)
% (*n*) detection of prostate cells		
CPCs	62.7% (116)	100% (30)
DTCs	62.2% (115)	100% (30)
mM	71.4% (132)	100% (30)

IQR: interquartile range, CPCs: circulating prostate cells, and DTC: disseminated tumor cells.

**Table 2:** Demographic variables according to Gleason score.

	Gleason 4	Gleason 5 + 6	Gleason 7	Gleason 8 + 9	*P* = (statistical test)
No. of patients (*n*)	28	106	31	20	
Mean age ± SD (years)	71.1 ± 8.7	73.2 ± 9.4	70 ± 8.9	71.7 ± 6.7	NS (ANOVA)
Median serum PSA (IQR) ng/mL	1.0 (0.5–4.8)	1.68 (0.5–5.5)	0.57 (0.1–10.0)	1.68 (0.32–28.7)	NS (Kruksal-Wallis)
Median stage (IQR)	2 (1-2)^a,b,c^	3 (2-3)^a,d^	3 (2-3)^b^	3 (3-4)^c,d^	a-a < 0.001 b-b < 0.001 c-c < 0.001 d-d < 0.002 Kruksal-Wallis (significant <0.004)
Median time from diagnosis (IQR) (years)	2 (1–4)	4 (1–8)	3 (1–5)	2 (1–5)	NS (Kruksal-Wallis)
Detection prostate cells % (*n*)					Chi-squared Log regression
CPC	46.4% (13)	63.2 (67)	64.5 (20)	80 (16)	*P* = 0.0123
DTC	35.7% (10)	65.1(69)	67.7 (21)	75 (15)	*P* = 0.015
mM	32.1% (9)	77.4 (82)	77.4 (24)	85 (17)	*P* = 0.001

IQR: interquartile range, CPC: circulating prostate cell, DTC: disseminated tumor cell, mM: micrometastasis, and NS: not significant.

**Table 3:** Demographic variables according to Gleason score in men with metastastic disease.

	Gleason 4	Gleason 5 + 6	Gleason 7	Gleason 8 + 9	*P* = (statistical test)
No. of patients (*n*)	1	13	11	5	
Mean age ± SD (years)	75.1 ± 7.7	74.2 ± 8.7	75 ± 7.2	74.2 ± 6.5	NS (ANOVA)
Median serum PSA (IQR) ng/mL	26	29 (19.0–150)	31 (22–150.0)	30 (19–150)	NS (Kruksal-Wallis)
Median stage at diagnosis	3	3	3	3	NS (Kruksal-Wallis)
Median time from diagnosis (IQR) (years)	8	8 (5–11)	7 (6–9)	7 (4–9)	NS (Kruksal-Wallis)

IQR: inter-quartile range, CPC: circulating prostate cell, DTC: disseminated tumor cell mM: micrometastasis, and NS: not significant.

**Table 4:** Frequency of MMP-2 expression in CPCs, DTCs, and mM in patients with nonmetastatic disease.

No. of patients	Gleason 4	Gleason 5 + 6	Gleason 7	Gleason 8 + 9	*P* = (statistical test, log regression)
Total 100% (*n* = 185)	15.1% (28)	57.3% (106)	16.8% (31)	10.8% (20)	
CPC positive 62.7% (*n* = 116)	46.4% (13)	63.2% (67)	64.5% (20)	80.0% (16)	NS
MMP-2	100% (13)	100% (67)	100% (20)	100% (16)	NS
DTC positive 62.5% (*n* = 115)	35.7% (10)	65.1% (69)	67.7% (21)	75.0% (15)	
MMP-2	100% (10)	100% (69)	100% (21)	100% (15)	NS
mM positive 71.4% (*n* = 132)	32.1% (9)	77.4% (82)	77.4% (22)	85% (17)	
MMP-2	0%^a,b,c^	14.6%^a,d^ (11)	20.8%^b^ (5)	41.1%^c,d^ (7)	a-a < 0.002 b-b < 0.002 c-c < 0.002 d-d < 0.002 Trend chi squared *P* = 0.031

CPC: circulating prostate cell, DTC: disseminated tumor cell, mM: micrometastasis, and NS: not significant.

**Table 5:** Concordance between the expression of MMP-2 in CPCs, DTCs, and mM according to Gleason score.

Kappa: MMP-2	Gleason 4	Gleason 5 + 6	Gleason 7	Gleason 8 + 9
CPC + DTC	0.64	0.59	0.78	0.57
CPC + mM	0	0.14	0.19	0.23
DTC + mM	0	0.13	0.17	0.30

Kappa values: 0–0.2 no concordance, 0.21–0.40 low concordance, 0.41–0.60 moderate concordance, 0.61–0.8 good concordance, >0.80 excellent concordance.

**Table 6:** Expression of MMP-2 in mM and surrounding stromal cells in patients with MMP-2 expressing mM.

No. of patients	Gleason 4	Gleason 5 + 6	Gleason 7	Gleason 8 + 9	
mM positive 71.4% (*n* = 132)	32.1% (9)	77.4% (82)	77.4% (22)	85% (17)	
MMP-2 in mM	0%	14.6% (11)	20.8% (5)	41.1% (7)	Trend chi squared *P* = 0.031
MMP-2 in stromal cells	0%	0%	4.5% (1)	11.8% (2)	
